# Effects of passive smoking on cortical spreading depolarization in male and female mice

**DOI:** 10.1186/s10194-024-01867-3

**Published:** 2024-10-02

**Authors:** Tsubasa Takizawa, Keiko Ihara, Miyuki Unekawa, Chisato Iba, Shizuko Kagawa, Narumi Watanabe, Shingo Nakayama, Kaori Sakurai, Naoki Miyazaki, Noriyuki Ishida, Ryo Takemura, Mamoru Shibata, Yoshikane Izawa, Shotaro Chubachi, Koichi Fukunaga, Jin Nakahara

**Affiliations:** 1 Department of Neurology, Keio University School of Medicine, Tokyo, 160-8582 Japan; 2 Japanese Red Cross Ashikaga Hospital, Tochigi, Japan; 3 Division of Pulmonary Medicine, Department of Medicine, Keio University School of Medicine, Tokyo, Japan; 4 Clinical and Translational Research Center, Keio University Hospital, Tokyo, Japan; 5 Department of Neurology, Tokyo Dental College Ichikawa General Hospital, Chiba, Japan

**Keywords:** Migraine with aura, Cortical spreading depolarization, Smoking, Sex difference, Mice

## Abstract

**Background:**

Patients with migraine are typically advised to avoid passive smoking because it may aggravate headaches and other health conditions. However, there is insufficient high-quality evidence on the association between passive smoking and migraine, which warrants further investigation using animal models. Therefore, using a mouse model, we examined the effect of passive smoking on susceptibility to cortical spreading depolarization (CSD), the biological basis of migraine with aura.

**Findings:**

Fifty C57BL/6 mice (25 males and 25 females) were exposed for one hour to cigarette smoke or room air. Subsequently, potassium chloride (KCl) was administered under isoflurane anesthesia to induce CSD, and the CSD threshold, frequency of induction, and propagation velocity were determined. The threshold to induce CSD (median [interquartile range (IQR)]) was significantly lower in female mice (adjusted *p* = 0.01) in the smoking group (0.05 [0.05, 0.088]) than in the sham group (0.125 [0.1, 0.15]); however, there was no significant difference in the male mice (adjusted *p* = 0.77). CSD frequency or propagation velocity did not differ significantly between the two groups for either sex.

**Conclusions:**

Female mice in the smoking group showed lower CSD threshold compared to the sham group, suggesting a potential sex-specific difference in the effect of smoking on the pathogenesis of CSD and migraine with aura. This finding may contribute to the understanding of migraine pathophysiology in association with passive smoking and sex difference.

## Introduction

Migraine, a significant public health problem that affects over 1 billion people worldwide [1, 2], ranks as the second leading cause of years of life lived with disability [3, 4]. Despite advancements in migraine medication, including anti-calcitonin gene-related peptide monoclonal antibodies (anti-CGRP mAbs) and gepants, only about 10% of patients can achieve complete control of attack frequency with drug therapy [5]. Therefore, physicians educate patients to adopt lifestyle modifications such as appropriate sleep, exercise, reduced alcohol intake, and avoiding passive smoking to manage migraine symptoms effectively.

Despite many studies reporting potential relationships between smoking and migraine onset [6] and recent reports linking secondhand smoke exposure to headaches and migraine in the nonsmoking population [7], the absence of high-quality evidence in humans prevents a consensus from developing regarding the existence of a causal relationship between migraine and passive smoking [6]. Given the established risks of smoking to humans, including increased risks of cancer and cardiovascular events [8], it is considered unethical to investigate the effect of smoking on migraine using human participants. Therefore, investigating the association between migraine and smoking through animal models of migraine might enhance our understanding of migraine pathophysiology.

Cortical spreading depolarization (CSD) is an established rodent model used to study migraine, especially migraine with aura. CSD is caused by the depolarization of neurons and glial cells, propagates through the cortex, and is a biological event occurring during the aura phase of migraine [9, 10]. Prior research has demonstrated that CSD was exacerbated by migraine-inducing or aggravating factors, including stress, and was suppressed by migraine prophylaxis [9, 11, 12].

We aimed to capture the potential effect of passive smoking on migraine with aura in an in vivo migraine model of CSD, considering the advantages of objective measurements in CSD.

## Methods

### Ethics

All experimental procedures were approved by the Keio University Institutional Animal Care and Use Committee (authorizations A2021-006 and A2022-257). All procedures were performed in accordance with university guidelines and Animal Research: Reporting of In Vivo Experiments (ARRIVE) reporting guidelines for the care and use of laboratory animals.

### Animals

Fifty C57BL/6 mice (25 males and 25 females, 8 weeks old) were purchased from CLEA Japan Inc. (Tokyo, Japan). They were housed for acclimatization under a 12-h dark–light cycle in an air-conditioned room (temperature of 23.0 °C ± 1.0 °C and humidity of 55% ± 7%) with unrestricted access to water and food for > 1 week before conducting the following experiments.

Of the 25 male and 25 female mice, the following animals were excluded from the analysis: Male mice, 2 due to death during exposure to cigarette smoke and 1 due to insufficient sample volume for blood gas analysis in the smoking group, 2 due to poor blood gas, and 1 due to a failure of femoral artery catheterization in the sham group; female mice: 2 due to death during smoke exposure, 1 due to poor blood gas, and 1 due to insufficient sample volume for blood gas analysis in the smoking group, 3 due to poor blood gas, 1 due to insufficient sample volume for blood gas analysis, and 1 due to ear bleeding in the sham group. Thus, 7–10 mice from each group (male-smoking, male-sham, female-smoking, and female-sham) were analyzed.

### Cigarette smoke exposure procedure

The mice were randomly assigned to either smoking or sham groups. Mice in the smoking group were restrained in an immobilization tube and exposed to cigarette smoke from commercially available filtered cigarettes (Marlboro, 12 mg tar/1.0 mg nicotine, Philip Morris, Richmond, VA) through nasal inhalation for 1 h, as previously reported. Following dilution with compressed air, the mass concentration of total particulate matter was 1,202 ± 196 mg/m^3^ [13]. Mice in the sham group were restrained in the same setting as the smoking group for immobilization and exposed to room air. One hour was allowed for recovery after exposure to cigarette smoke or room air so that the physiological state could remain unchanged during the recordings.

### CSD evaluation

**Fig. 1:**
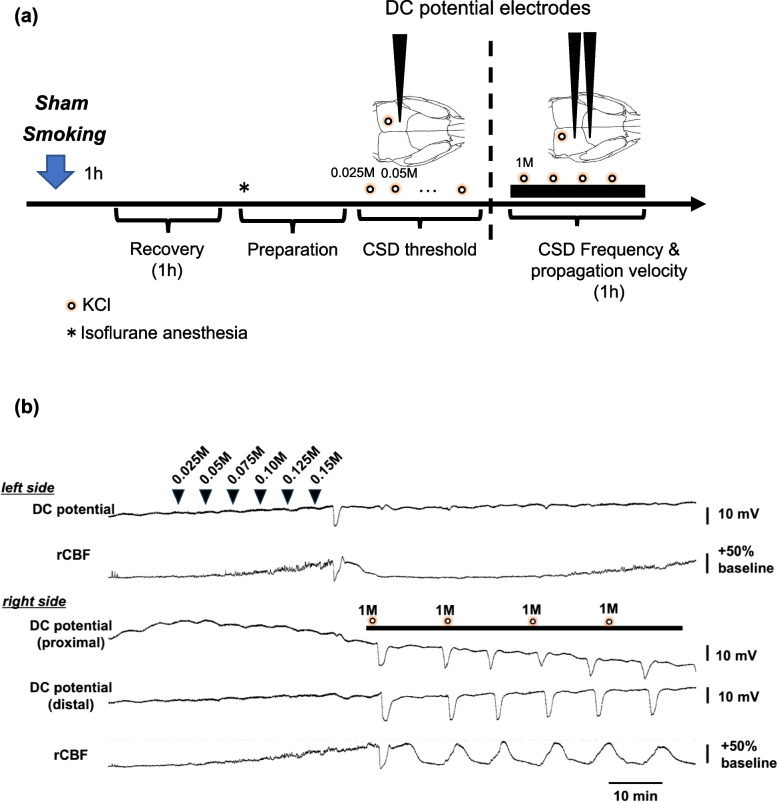
**a** Experimental protocol and CSD setup. Fifty C57BL/6 mice (25 males and 25 females) were divided either into the smoking group to receive cigarette smoke exposure for one hour (1 h) or the sham group to receive room air for 1 h in a tube for immobilization. After 1 h of recovery, followed by preparation, the minimum KCl concentration to evoke CSD (threshold of CSD induction) and frequency of CSD induced by a 1 M KCl cotton ball were measured in 1 h. The propagation velocity was calculated from the time differences and distances between two direct current (DC) electrodes. **b** Representative raw tracing of recorded CSD. DC, direct current; rCBF, regional cerebral blood flow

The following parameters were recorded: direct current (DC) potential, regional cerebral blood flow (rCBF), systemic arterial blood pressure (ABP), and heart rate (HR). The rectal temperature was maintained at 37 °C with a heating pad and thermocontroller (BWT-100, Bio Research Center Co. Ltd., Nagoya, Japan) as described previously [14] (Fig. 1-(a), (b)). In brief, one hour after smoking or being restrained, a catheter was inserted into the femoral artery to monitor blood pressure under isoflurane anesthesia (2%–2.3%). The mouse was secured to a head-holder (SGM-4, Narishige Scientific Instrument Laboratory, Tokyo, Japan). Prior to CSD evaluation, arterial blood was drawn through the catheter for blood gas analysis with RapidLab 348EX (Siemens AG., Munich, Germany). DC potentials were monitored using three electrodes (EEG-5002Ag; tip diameter = 200 µm; Bio Research Center) that were fixed in the bilateral parietal (± 2 mm lateral and 2 mm caudal to the bregma) and right frontal (2 mm lateral and 1 mm rostral to the bregma) openings in the skull. Over the parietal bone, rCBF was monitored bilaterally using laser Doppler flowmeters (ALF21, Advance Co. Ltd., Tokyo, Japan). Continuous recordings of DC potential, rCBF, ABP, and HR were stored on a multichannel recorder (PowerLab 8/35; ADInstruments Pty Ltd., Sydney, Australia). The LabChart software (ADInstruments Pty Ltd.) was used for offline analysis (Fig. 1-(b)). We considered it a CSD occurrence when we observed unilateral CBF changes that were characteristic of CSD and were synchronized with the deflection of DC potentials. DC potentials were recorded with monopolar electrodes, with a reference electrode placed subcutaneously in the dorsum.

After reducing the isoflurane concentration to 1.5% and confirming that no CSD occurred for a duration exceeding 30 min, 5 µL of 0.025 M potassium chloride (KCl) solution was introduced into the left posterior hole; the KCl concentration was increased in a stepwise manner by 0.025 M at intervals of at least 5 min. The concentration at which CSD first manifested was designated as the threshold for CSD [14, 15]. CSD induction was validated by the presence of characteristic DC potential deflection and unilateral rCBF fluctuation. The frequency of CSD induced in the right hemisphere was assessed by calculating the number of CSD events that occurred within a 60-min period. The 1-M KCl cotton ball was replaced every 15 min. The propagation velocity of the first-manifested CSD was calculated using the distance and time difference between the two electrodes. CSD measurement was performed in part by a blinded investigator.

### Statistics

We analyzed the threshold to induce CSD, the frequency of CSD at 1 M KCl, and the propagation velocity of CSD (Fig. 1). Animals that were deceased or exhibited poor physiological status, or in which technical errors occurred during the procedure were excluded. In general, we used the Wilcoxon rank sum test. The Wilcoxon rank sum test with Bonferroni correction was used for the statistical analysis of the effect on the CSD threshold. Statistical analysis was performed using the SAS version 9.4 software (SAS Institute, Cary, NC), and graphs were created using the Prism version 9.2.0 software (GraphPad Software, La Jolla, CA).

## Results

**Fig. 2:**
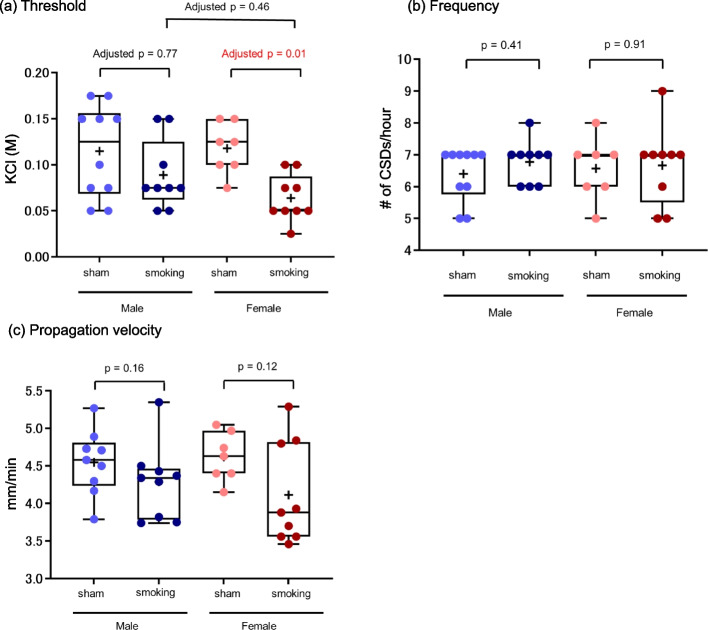
**a** Threshold, **b** Frequency, and **c** Propagation velocity of CSD between the smoking and sham groups in male and female mice. Whiskers represent the complete range; boxes represent the interquartile range; horizontal lines represent the median; and + represents the mean. Individual data points from each animal are also shown

In the female mice, the median [interquartile range (IQR)] of the induction threshold was significantly lower in the smoking group (0.05 [0.05, 0.088]) than in the sham group (0.125 [0.1, 0.15]) (adjusted *p* = 0.01). Overall, other measurements yielded nonsignificant results. The median threshold of CSD in male mice was 0.075 [0.063, 0.125] in the smoking group and 0.125 [0.069, 0.156] in the sham group (adjusted *p* = 0.77) (Fig. 2). The adjusted p-value between the male and female mice in the smoking group was 0.46. There were no significant differences in the frequency of CSD induction or the propagation velocity between smoking and sham groups in both male and female mice (Fig. 2).

In the arterial blood analysis, pH was higher (*p* = 0.03, 0.03 in males and females, respectively), and [Na^+^] was lower (*p* = 0.02, *p* < 0.01 in males and females, respectively) in the smoking group. In the male mice, [HCO_3_^−^] and [K^+^] were higher in the smoking group (*p* < 0.01, *p* = 0.03). In the female mice, mean arterial blood pressure (MABP) was lower in the smoking group (*p* < 0.01), as shown in Table 1.

**Table 1:** Comparison of physiological parameters between the smoking and sham groups

	Male			Female		
	Smoking	Sham		Smoking	Sham	
n	9	10		9	7	
Age (weeks)	10.1 ± 0.8	10.4 ± 1.0		9.8 ± 0.8	9.6 ± 0.8	
Body weight (g)	23.5 ± 1.1	23.0 ± 1.2		18.2 ± 0.9	18.1 ± 1.3	
pH	7.39 ± 0.04	7.35 ± 0.04	*	7.39 ± 0.03	7.35 ± 0.03	*
PaCO_2_ (mmHg)	35.7 ± 4.9	31.1 ± 5.1		30.3 ± 3.2	32.4 ± 2.7	
PaO_2_ (mmHg)	99.6 ± 11.6	103.8 ± 9.8		112.8 ± 9.1	110.9 ± 5.9	
Na (mEq/L)	142.8 ± 2.1	145.9 ± 2.6	*	141.1 ± 2.6	145.0 ± 1.6	**
K (mEq/L)	4.93 ± 0.48	4.45 ± 0.40	*	4.36 ± 1.04	4.12 ± 0.14	
HCO_3_^−^ (mmol/L)	21.1 ± 1.6	16.7 ± 1.9	**	17.7 ± 1.4	17.5 ± 1.6	
MABP (mmHg)	78.3 ± 3.0	78.5 ± 6.8		76.3 ± 4.9	84.5 ± 4.6	**
HR (bpm)	464 ± 33	436 ± 47		443 ± 22	476 ± 68	

*MABP* mean arterial blood pressure, *HR* heart rate. Data indicate the mean ± standard deviation. The Wilcoxon rank sum test was performed for comparison between smoking and sham groups (*, *p* < 0.05; **, *p* < 0.01). Missing data were omitted for analysis (male-smoking: *n* = 8 for Na)

## Discussion

In this study, the median CSD threshold in female mice in the smoking group was significantly lower than that in the sham group. Cigarette smoke exposure might have predisposed the female mice to CSD, a biological event that occurs during the aura phase of a migraine and is thought to be involved in the pathogenesis of migraine with aura [16]. This sex difference could be discussed both in terms of sex difference in migraine pathophysiology and cigarette smoke susceptibility.

First, previous observational human studies have controversially linked cigarette smoking to migraine onset and triggers. A Danish population-based cross-sectional study on 31,865 twin individuals found that both women and men with a smoking history demonstrated increased risks for both migraine with aura and migraine without aura [17]. Norwegian large-scale population-based cross-sectional studies revealed that smokers were more likely to have migraine [18, 19], but did not elucidate the relevant data regarding aura and sexes. A prospective study of 54 patients revealed that the greatest risk factor for the occurrence of aura is smoking; however, smoking decreased the risk of headache or migraine without aura [20]. Regarding differences between sexes, a survey of 361 Spanish medical students revealed that the prevalence of active smoking is one-third higher in students with migraine. A clearer relationship between migraine and smoking was observed in females, partly owing to the lesser number of males among both medical students and smokers [21]. Additionally, a retrospective study on 494 patients with migraine in the US revealed that 26% of them cited cigarette smoke as a trigger factor, with the proportion of females (29%) being significantly higher compared to males (13%) [22]. The difference related to aura was not clarified in either of these two studies. Moreover, a literature review revealed inconclusive results regarding the association between smoking and headaches [6]. These conflicting findings warrant further investigation to fully understand the association between migraine and smoking in both humans and animals.

Second, susceptibility to cigarette smoke in the respiratory system has been considered sex-dependent and often more severe in females, both in mice [23] and human [24, 25]. The assumably greater injury of the respiratory systems by cigarette smoke might have predisposed female mice brain to be affected to a greater extent in our study.

Previous evidence regarding cigarette smoke can be utilized to analyze the potential mechanisms underlying the decreased threshold of CSD in this study. Cigarette smoke contains more than 4,700 chemicals, with varying percentages among different cigarette types [6]. Specifically, nicotine and carbon monoxide (CO) are recognized to affect the nervous system. For example, stimulation of nicotinic receptors increases the sensitivity of the chicken retina to retinal spreading depolarization [26]. This may be similar to the mechanism of the CSD sensitivity change induced by smoking in this study. Another study exhibited that CO exposure influences the frequency of CSD in rat brains; however, the impact of CO on CSD frequency varied depending on the time after exposure [27]. For instance, CSD frequency decreased immediately after CO exposure but increased 90 min later. Notably, the study was conducted exclusively on male rats; therefore, sex and species differences must be considered when interpreting the results.

Interestingly, in contrast to a previous study that showed a significantly reduced threshold for induction of CSD in female mice compared to male mice [28], our results did not show any significant difference between females and males in CSD threshold in the sham group. This discrepancy may be due to the differences in experimental setups, and further studies with larger sample sizes would be necessary to conclude this topic.

Regarding blood gas analysis and MABP, we observed several significant differences between the two groups. The smoking group exhibited elevated pH levels and decreased [Na^+^] in both sexes. Elevated pH may have potentially affected CSD parameters; since NMDA receptors are inhibited by extracellular protons [29], increased pH may have possibly decreased the tonic proton block and increased cortical excitability. Hyponatremia is associated with increased cortical excitability [30] and may also have affected CSD parameters. Only male mice in the smoking group had elevated [K^+^] and [HCO3^−^] concentrations. Female mice exhibited lower MABP in the smoking group. MABP was previously shown to be negatively correlated with CSD durations [31], but we did not observe a significant change in CSD duration. Currently, there is no established evidence of the association between blood gas analysis or MABP and the threshold of CSD [32]. Therefore, we cannot determine whether these factors influenced the changes in CSD threshold in female mice. Further research is required to examine the correlations.

The study has a few limitations. The acute effects of smoking on CSD were investigated in this study; therefore, the discussion may not be applicable to the chronic effects of smoking. Because exposure to smoking was conducted in only one condition, the dose–effect relationship of smoking could not be determined. Another limitation is that the natural estrous cycle could have contaminated our results; a previous study showed that the CSD threshold was significantly reduced during the diestrus period [33]. However, this possible effect of the estrous cycle on our results was minimized in our randomized controlled study design using 7–9 mice per group. Moreover, to conclude our question, additional quantitative data and mechanism studies are necessary. Lastly, as CSD is an animal model of migraine with aura, the results cannot be directly extrapolated to migraine without aura.

## Conclusion

Female mice in the smoking group showed lower CSD threshold compared to the sham group. We suggest that there may be sex-based differences in the impact of smoking on migraine via a stronger predisposition to CSD in female mice. Additional research is needed to verify if smoking cessation could be beneficial in preventing or managing migraine.

## Data Availability

The datasets used and/or analyzed during the current study are available from the corresponding author upon reasonable request.

## Abbreviations

CSD: Cortical spreading depolarization
KCl: Potassium chloride
CO: Carbon monoxide
MABP: Mean arterial blood pressure
ABP: Arterial blood pressure
DC: Direct current
rCBF: Regional cerebral blood flow
HR: Heart rate
